# Holes Outperform Electrons in Group IV Semiconductor Materials

**DOI:** 10.1002/smsc.202200094

**Published:** 2023-03-02

**Authors:** Maksym Myronov, Jan Kycia, Philip Waldron, Weihong Jiang, Pedro Barrios, Alex Bogan, Peter Coleridge, Sergei Studenikin

**Affiliations:** ^1^ Physics Department The University of Warwick Coventry CV4 7AL UK; ^2^ Physics and Astronomy Department University of Waterloo Waterloo N2L 3G1 Canada; ^3^ Security and Disruptive Technologies Research Centre National Research Council of Canada Ottawa K1A 0R6 Ontario Canada

**Keywords:** 2D hole gases, germanium, mobility, quantum materials, semiconductors, spin orbit interaction

## Abstract

A record‐high mobility of holes, reaching 4.3 × 10^6^ cm^2^ V^−1^ s^−1^ at 300 mK in an epitaxial strained germanium (s‐Ge) semiconductor, grown on a standard silicon wafer, is reported. This major breakthrough is achieved due to the development of state‐of‐the‐art epitaxial growth technology culminating in superior monocrystalline quality of the s‐Ge material platform with a very low density of background impurities and other imperfections. As a consequence, the hole mobility in s‐Ge appears to be ≈2 times higher than the highest electron mobility in strained silicon. In addition to the record mobility, this material platform reveals a unique combination of properties, which are a very large and tuneable effective g*‐factor (>18), a very low percolation density (5 × 10^9^ cm^−2^) and a small effective mass (0.054 *m*_0_). This long‐sought combination of parameters in one material system is important for the research and development of low‐temperature electronics with reduced Joule heating and for quantum‐electronics circuits based on spin qubits.

## Graphical abstract

A record‐high mobility of holes in germanium semiconductor grown on a standard silicon wafer is reported, which exceeds the previous state‐of‐the‐art value by over 4 times. Moreover, this material platform reveals a unique combination of properties, essential for quantum‐electronics applications, which are very large and tuneable effective g*‐factor, the lowest percolation density, and small effective mass.

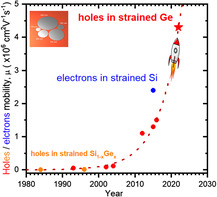

## 1 Introduction

The semiconductor industry is one of the largest industries in the world, with global sales of $555 billion in 2021.^[^ ^1^ ^]^ Over 99% of all semiconductor devices are made of or on silicon (Si) wafers. Novel group IV semiconductor epitaxial structures composed of silicon (Si), germanium (Ge), carbon and diamond (C), or tin (Sn) on silicon or silicon‐on‐insulator substrates provide a natural route for continued improvement of properties of modern state‐of‐the‐art Si devices with expanding functionalities for mass production. These materials underpin devices with new and/or enhanced properties for applications in electronics, optoelectronics, thermoelectrics, spintronics, sensors, and quantum electronics based on spin qubits.

Mobility of free carriers in conduction (electrons) or valance (holes) bands, along with a reasonably large energy bandgap, is one of the most important quality measures of any semiconductor material, determining its suitability for applications in a large variety of classical electronic, optoelectronic, and sensor devices, as well as for novel applications in emerging quantum devices. Higher mobility enables faster operation of a device at lower power consumption and thus leading to reduced Joule heat dissipation, which is essential for scaling and increasing the speed of current electronic devices. It is even more important for those devices and electronics, which work at cryogenic temperatures and are intended to control distributed registers of quantum processors.^[^ ^2^ ^]^ Also, carrier mobility is the critical quality for quantum devices, often playing a key role toward new discoveries.^[^ 3, 4, 5 ^]^

When one or more dimensions of a material are reduced sufficiently to the nanometer range, at a scale comparable to the de Broglie wavelength of the carriers, its properties become different from those of the bulk (3D) material. With reduction in size, novel electrical, mechanical, chemical, magnetic, thermal, optical, and other properties emerge. The resulting structure which could be either 2D, 1D, or 0D is then called a low‐dimensional structure or system. Compared to conduction band electrons, holes in the valance band possess more complex energy structure. This leads to many special properties including a reduced hyperfine interaction with nuclear spins that is useful for enhanced spin coherence in quantum devices, large and controllable spin–orbit interaction (SOI) for fast and locally addressable spin‐based qubits, controllable light–heavy hole interaction for the energy band and g* (effective g‐factor)‐factor engineering, etc.^[^ ^6^ ^]^ Compared to electrons, all these complex hole properties can be considered as additional resources for engineering new devices based on hole spins, on SOI, and now on superior mobility. This explains the recent fast‐growing interest in p‐type (i.e., hole) semiconductor materials for fundamental research and applications. Holes in strained Ge (s‐Ge) possess additional unique properties compared to all semiconductors, including p‐Si and p‐GaAs. In particular, they have much smaller effective mass, which can be engineered down to 0.035 *m*_0_,^[^ ^7^ ^]^ and a large electric‐field tuneable effective g*‐factor of up to 15.^[^ ^8^ ^]^ The small effective mass and large g*‐factor are essential properties for simplifying fabrication technology for quantum devices since larger orbital quantization and spin splitting energies enable larger lithographic features and higher‐operation temperatures. These properties are also attractive for the development of topological quantum devices employing Majorana fermions.^[^ ^9^ ^]^ One more significant property should be emphasized: Ge is a centrosymmetric elemental semiconductor, and therefore does not inherit the large bulk Dresselhaus component of SOI that would arise from bulk inversion asymmetry.^[^ ^10^ ^]^ The Dresselhaus bulk SOI is very strong in all III–V compound semiconductors causing additional spin–orbit (SO)‐mediated decoherence processes that are harmful for spin qubit devices. In contrast, SO is a useful interaction mechanism if it could be controlled. This is exactly the case for s‐Ge devices with structural inversion asymmetry in the growth direction. Indeed, only the Rashba SO component remains active in s‐Ge structures, which can be engineered and controlled by a gate voltage. For example, it can be completely switched off or quickly turned on at desired moments for spin manipulations and quantum‐information processing.^[^ 10, 11 ^]^ This is a very advantageous property of Rashba‐type SOI compared to the Dresselhaus coupling, because the coupling parameter describing strength of the Dresselhaus spin–orbit forces is a bulk material constant (tensor) and cannot be tuned by external electric fields.

Semiconductor heterostructures have a built‐in strain that is induced by the mismatch of the crystal lattices of the composed materials. It is an essential parameter used for energy band structure engineering of the heterostructures based on various semiconductors, including Si and Ge. Development of high‐mobility‐strained Si, SiGe, and Ge quantum‐well (QW) heterostructures requires special growth techniques—molecular beam epitaxy (MBE) and chemical vapor deposition (CVD)—combined with epitaxial technologies to overcome various challenges in heteroepitaxy of these materials. Over the past decades, progress in epitaxial technologies have led to achieving record‐high 2D hole gas (2DHG) mobilities in s‐Ge QW modulation‐doped (MOD) heterostructures, epitaxially grown on a standard Si(001) substrate, at both cryogenic and room temperatures.^[^ 4, 12, 13, 14, 15 ^]^ The mobility range of 2DHGs at room temperature was extended by 50% to 4500 cm^2^ V^−1^ s^−1^.^[^ ^13^ ^]^ This value outperforms the room‐temperature 2DHG mobility of any known *epitaxially grown* semiconductors with nonzero bandgap, including III–V, and single‐layer 2D materials. At lower temperatures, that is, below 10 K, much higher 2DHG mobilities in the range of 100 000–1 300 000 cm^2^ V^−1^ s^−1^ have been demonstrated.^[^ 7, 12, 15, 16, 17, 18, 19, 20, 21 ^]^ It is interesting to note that these high 2DHG mobilities at both low and room temperatures were not predicted theoretically.

**Figure 1:**
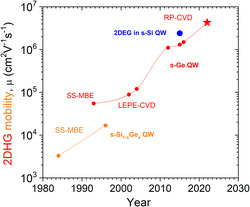
The historic evolution of the 2DHG mobility in the group IV semiconductors at low temperatures. The star marks result of this work. The highest 2DEG mobility in s‐Si quantum well (QW) is shown for comparison (blue solid point).

The historic evolution of 2DHG mobility in the group IV semiconductors at low temperatures is shown in **Figure** **1**.^[^ ^22^ ^]^ The first MOD‐strained Si_1−*x*_Ge_*x*_ (s‐Si_1−*x*_Ge_*x*_) QW heterostructures reported in 1984 were grown by solid‐source MBE (SS‐MBE) and had a 2DHG mobility of just 3300 cm^2^ V^−1^ s^−1^.^[^ 23, 24 ^]^ By 1996, this value was improved up to 16 800 cm^2^ V^−1^ s^−1^ in a low‐Ge‐content Si_0.87_Ge_0.13_ QW.^[^ ^25^ ^]^ The large lattice mismatch between Ge and Si (4.17% at 293 K) made it impossible to grow QW heterostructure with high Ge content coherently on a Si substrate. For this reason, relaxed Si_1−*x*_Ge_*x*_ buffers were developed and utilized to produce high‐quality s‐Ge structures on Si. By 1993, the highest 2DHG mobility in s‐Ge QW heterostructure was 55 000 cm^2^ V^−1^ s^−1^ and the material was grown by SS‐MBE.^[^ ^25^ ^]^ The next improvement was achieved by using the low‐energy plasma‐enhanced CVD (LEPE‐CVD). This growth technology was able to enhance hole mobility by a factor of ≈2 up to ≈100 000 cm^2^ V^−1^ s^−1^, by 2002.^[^ ^26^ ^]^ Development of reduced‐pressure CVD (RP‐CVD) provided a breakthrough in 2012, resulted in a ×10 enhancement of 2DHG mobility up to ≈1 000 000 cm^2^ V^−1^ s^−1^.^[^ 12, 27 ^]^ Finally, 10 years later and 38 years since the invention of the Si_1−*x*_Ge_*x*_ QW heterostructures, the results reported here present another long‐awaited major breakthrough in achieving the next record of hole mobility in s‐Ge QW of 4.3 × 10^6^ cm^2^ V^−1^ s^−1^. This is over ≈3 times higher than the previously reported record of ≈1.3 × 10^6^ cm^2^ V^−1^ s^−1^ in s‐Ge QW.^[^ ^7^ ^]^ This remarkable breakthrough in mobility can be viewed as an emergence of new class of quantum materials based on s‐Ge with unique spin properties reported here. For comparison, in Figure 1, we also show the highest mobility of a 2D electron gas (2DEG) in tensile‐strained Si (s‐Si) QW.^[^ ^28^ ^]^ This is the first time that electrons are outperformed by holes in a group IV semiconductor material at low temperatures. Currently, the record hole mobility is twice that of electrons for this material. We are unaware of a similar situation for any other semiconductor. Electron mobility has always been higher than that of holes in other semiconductors.

Enhanced electron and hole mobilities are not only important for developing novel electronic devices, but also open opportunities for new research in nanostructures that may lead to discoveries of new quantum phenomena. Moreover, very low‐effective mass of 2DHG in s‐Ge QW^[^ ^7^ ^]^ facilitates the realization of laterally gated quantum devices such as quantum‐point contacts (QPC), 1D wires, and quantum‐dot (QD) devices,^[^ ^29^ ^]^ operating at milli‐Kelvin or up to 4.2 K.^[^ ^4^ ^]^ High mobility at small carrier densities along with small effective mass and large effective g*‐factor of the s‐Ge‐based materials enables fabrication of reproducible arrays of quantum devices with designer properties that can be reliably predicted. Moreover, the fact is that the devices based on the planar 2DHG structures with relatively small effective mass have relatively large lateral dimensions and, therefore, possess pure 2D characteristics determined by the hole energy spectrum originated from quantization in the vertical, that is, growth, direction of the heterostructure means that such properties can be engineered.^[^ ^5^ ^]^

## 2 Results and Discussions

### 2.1 Epitaxial Growth of s‐Ge QW Heterostructures

**Figure 2:**
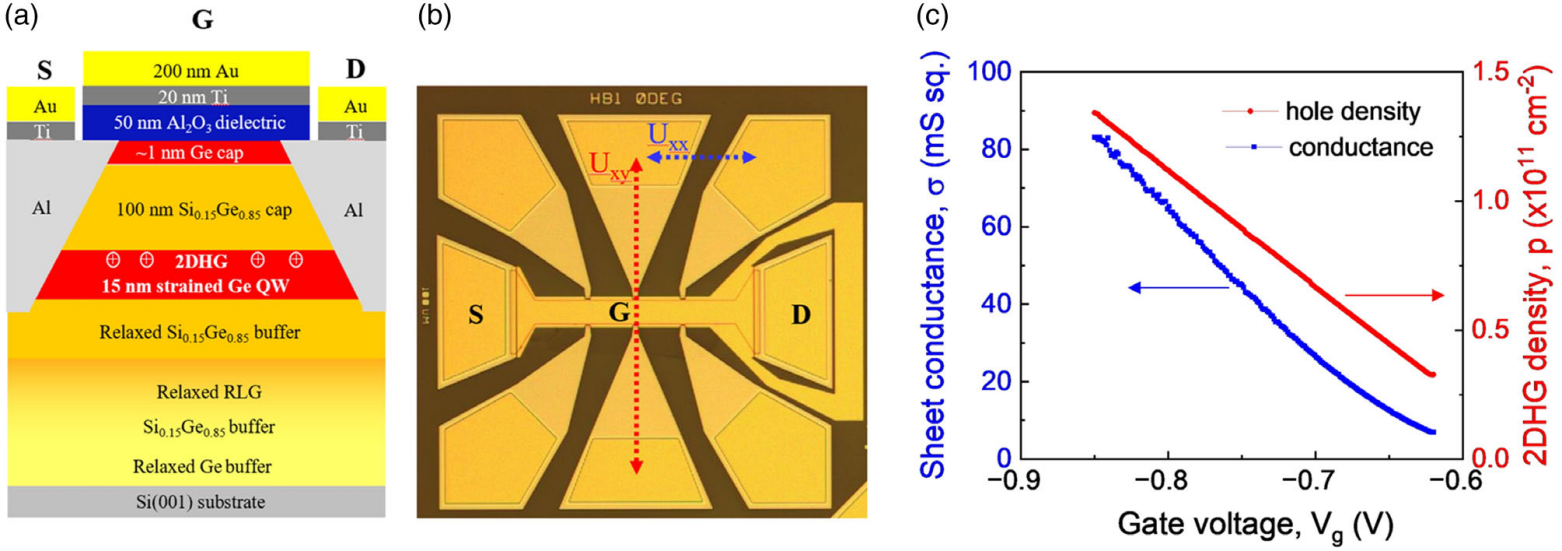
a) Schematic cross section of the s‐Ge QW heterostructure used to fabricate the gated Hall‐bar device. b) A microscopy image of a gated Hall‐bar with the channel width, *w*, of 100 μm and the distance between the nearest potential contacts, L, of 200 μm. c) Gate‐voltage‐dependence.

For the presented research, an undoped s‐Ge QW heterostructure was grown by RP‐CVD on a relaxed Si_0.15_Ge_0.85_ buffer on a standard Si(001) wafer of 150 mm diameter.^[^ 7, 27 ^]^ A schematic cross section of the heterostructure and fabricated gated Hall‐bar device, with its source (S) and drain (D) Ohmic contacts and gate (G) stack, is shown in **Figure** **2**a.^[^ 7, 27 ^]^ Epitaxy of the relaxed buffer layer and active region of a 15 nm‐thick s‐Ge QW were carefully optimized to substantially improve the material quality. In particular, the growth temperature of the s‐Ge QW region was below 500 °C to suppress the Si and Ge interdiffusion, Ge segregation and strain relaxation in the s‐Ge QW. Both Si and Ge precursors were additionally purified to suppress background contamination. All epilayers were intentionally undoped. Accumulated by the negative gate voltage, holes are confined in the 15 nm‐thick undoped QW, positioned 100 nm below the Si_0.15_Ge_0.85_ cap epilayer and ≈1 nm‐thick Ge cap interface. A thick Si_0.15_Ge_0.85_ cap epilayer was intentionally selected to minimize impact of the remote ionized impurities located at the Ge cap/Al_2_O_3_ gate dielectric and within the gate dielectric.^[^ ^8^ ^]^ A ≈1 nm Ge cap epilayer thickness is expected to remain after the surface cleaning of an initially thicker Ge cap, prior to the deposition of the gate dielectric. Absence of a boron MOD impurity layer eliminates the presence of ionized doping impurities in close vicinity to the 2DHG confined in the s‐Ge QW.^[^ ^22^ ^]^ Omitting the in situ doping process also eliminates any unintentional boron doping due to either diffusion of impurities or their segregation in the case of inverted MOD.^[^ ^7^ ^]^ Scattering on both remote and background ionized impurities is responsible for limiting 2DHG mobility at low temperatures. In our heterostructure, we achieved a very small level of ionized background impurities, resulting in the new benchmark record of 4.3 × 10^6^ cm^2^ V^−1^ s^−1^ for hole mobility in the group IV semiconductor material.

### 2.2 Gated Hall‐Bar Devices Microfabrication

For transport characterization of 2DHG of varied density, gated Hall‐bars were fabricated using standard UV lithography, dry etching, and thin‐film deposition techniques. Figure 2b shows a typical optical microscope image of a Hall bar with its channel oriented along the ⟨110⟩ in‐plane crystallographic direction^[^ ^7^ ^]^ and defined by the mesa structure etched in Cl_2_/Ar plasma. The Hall‐bar's channel width is 100 μm and the distance between the nearest potential contacts is 200 μm. The alloyed AlSiGe Ohmic contacts are prepared by evaporating a 120 nm‐thick Al film and then annealing at ≈275 °C in N_2_ ambient for 30 min. The contacts show low resistivity and excellent linear Ohmic behavior at cryogenic temperatures. The top accumulation gate is made of 20 nm Ti followed by 200 nm Au on a 50 nm‐thick Al_2_O_3_ dielectric layer deposited by atomic layer deposition (ALD) at 200 °C. Figure 2c shows how the variation of applied gate voltage from approximately −0.6 up to −0.85 V at 290 mK induces changes to the 2DHG density and sheet conductivity from ≈0.32 × 10^11^ to ≈1.4 × 10^11^ cm^−2^ and from ≈10 to ≈90 mS sq^−1^, respectively. The linear dependence of the hole density versus gate voltage has no noticeable hysteresis that indicates a relatively good quality Al_2_O_3_ dielectric and an interface with a low density of rechargeable traps.

### 2.3 Magnetotransport Characterization

The gated Hall‐bar was mounted in a ^3^He cryostat equipped with a superconducting solenoid. For the low‐temperature transport measurements, we employed a standard lock‐in technique with an excitation current, *I*, of 20 nA limited by a 1 MΩ in‐series resistor. We used to two synchronized lock‐ins at 88 Hz to measure longitudinal (*U*_*xx*_) and transverse or Hall (*U*_*xy*_) voltages simultaneously. Zero magnetic‐field resistivity, *ρ*_0_ = *wU*_*xx*_/*IL*, and the Hall voltage in a small non‐quantizing magnetic field are used to calculate carrier density $p=B/eR_{xy}$ of 2DHG and carrier‐transport mobility, $\mu =R_{xy}\left(B\right)/B\rho_{xx}\left(0\right)$. A gate voltage applied between the gate *G* and the drain *D*, in Figure 2b, is used to control the 2DHG density and conductance of the s‐Ge QW channel.

### 2.4 Mobility

**Figure 3:**
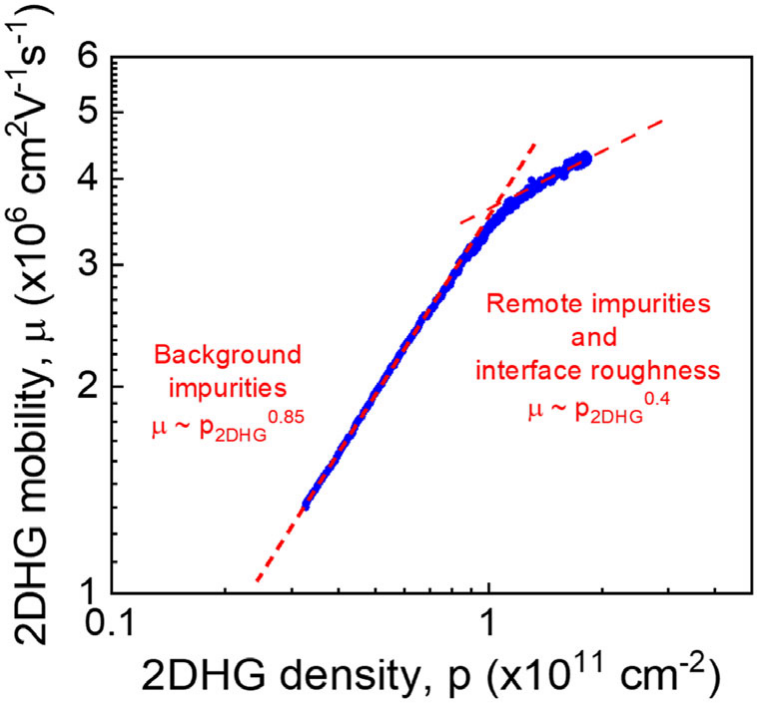
Carrier mobility versus 2D hole gas (2DHG) density measured at the base temperature of 290 mK.

The experimentally obtained 2DHG mobility as a function of the 2DHG density is plotted on log–log scale in **Figure** **3**. The 2DHG mobility varies from 1.2 × 10^6^ cm^2^ V^−1^ s^−1^ at 2DHG density of 3.2 × 10^10^ cm^−2^ to 4.3 × 10^6^ cm^2^ V^−1^ s^−1^ at 1.8 × 10^11^ cm^−2^. The mean free path of holes for these data increases from ≈5 μm in low density range and reaches microscopic magnitudes up to 30 μm for higher densities. The maximum mobility of 4.3 × 10^6^ cm^2^ V^−1^ s^−1^ exceeds by over 3 and 4 times the previously reported highest values of ≈1.3 × 10^6^ cm^2^ V^−1^ s^−1^ at *p* = 2.7 × 10^11^ cm^−2^ in MOD s‐Ge QW heterostructure^[^ ^7^ ^]^ and ≈1 × 10^6^ cm^2^ V^−1^ s^−1^ at 1 × 10^11^ cm^−2^ in undoped gated s‐Ge QW heterostructure,^[^ ^30^ ^]^ respectively. Analysis of the mobility versus carrier density slope indicates the mobility at lower carrier density range, that is, below 1 × 10^11^ cm^−2^ is limited by scattering on background ionized impurities with an estimated volume density of ≈3 × 10^14^ cm^−3^.^[^ ^31^ ^]^ This very low background impurity density is manifested as an exceptionally high mobility in the whole range of 2DHG densities, that is, the sample show record‐high 2DHG peak mobility 4.3 × 10^6^ cm^2^ V^−1^ s^−1^ in the high density range of ≈1.8 × 10^11^ cm^−2^, and also very high mobility over 1.2 × 10^6^ cm^2^ V^−1^ s^−1^ in the low density range down to 3.2 × 10^10^ cm^−2^, seen in Figure 3. Fitting of the experimental data in low‐density range, that is, <1 × 10^11^ cm^−2^ shows an almost linear increase of the mobility with the density, that is, *μ *≈ *p* ^0.85^. At higher than 1 × 10^11^ cm^−2^ carrier density, the mobility increase slows down, following a power‐law dependence with a smaller exponent, *μ* ≈ *p* ^0.4^. This dependence indicates that hole mobility in the high‐density range is limited by some additional scattering mechanisms. Most likely, they are due to remote ionized impurities at the dielectric/semiconductor interface between the Al_2_O_3_ gate dielectric and the surface Ge cap layer; or/and interface roughness at the s‐Ge QW and Si_0.15_Ge_0.85_ barrier interface, or possibly other crystal imperfections.

Defects which can limit hole mobility in our 15 nm s‐Ge QW epilayer are threading dislocations originating in the relaxed buffer and propagating through the whole structure up to the surface. Our optimized relaxed buffer layer shows relatively low threading dislocations density (TDD) in the range of 10^6^ cm^−2^.^[^ 14, 27 ^]^ However, TDD may be responsible for the mobility limitations reported here. Assuming a random distribution, the previously mentioned TDD corresponds to ≈1 threading dislocation per 10 μm. This is comparable to the obtained transport mean free path of ≈30 μm. If it is indeed the case, then the only way to validate this hypothesis will require further developments of new generation of relaxed buffers with even lower TDD, that is, in the range of ≈10^4^–10^5^ cm^−2^ than the current state of the art reported here.

The s‐Ge QW/Si_0.15_Ge_0.85_ interface roughness could also be responsible for limiting the measured maximum mobility of 4.3 × 10^6^ cm^2^ V^−1^ s^−1^ at 1.8 × 10^11^ cm^−2^. There are two known origins of the interface roughness in an s‐Ge QW heterostructure. The first is the surface roughness of relaxed Si_0.15_Ge_0.85_/Ge buffer layer grown on the Si(001) substrate. It manifests itself by the appearance of a correlated cross‐hatched pattern on the surface. Such a pattern occurs as a consequence of strain relaxation due to the inhomogeneous distribution of misfit dislocations at different planes within the buffer. It is interesting to note that the period of these cross‐hatches is typically below 5 μm.^[^ ^27^ ^]^ Though, the surface of such a buffer is very smooth with the root‐mean‐square (RMS) surface roughness being about of ≈2 nm.^[^ ^27^ ^]^ This cross‐hatched roughness may also contribute to the scattering mechanisms limiting 2DHG mobility reported here. The second origin of the interface roughness is purely due to the smoothness and heteroepitaxy fluctuations of the s‐Ge QW active region. In our case, it was carefully optimized to maintain the interface to be smooth and abrupt. For a more detailed examination of the aforementioned mechanisms, we plan to conduct similar experiments on a series of QW structures with varied s‐Ge QW thickness.

We believe that the maximum mobility has still not been reached in s‐Ge QW heterostructures and there is room for further improvements. Clearly, more detailed experimental and theoretical studies are required to understand microscopic mechanisms that limit hole mobility in s‐Ge heterostructures. However, it is clear that the higher‐quality epitaxial growth provided by RP‐CVD is the key factor that contributed to obtaining ≈80 times higher 2DHG mobility in s‐Ge QW structure grown by RP‐CVD compared to the best one grown by SS‐MBE. This technology also results in improved quality of interfaces and a reduction in background ionized impurities and defects, not only in s‐Ge, but also in the surrounding relaxed SiGe epilayers of the heterostructure. The value of RP‐CVD technology becomes even clearer considering that growth pressures are relatively high at 10–100 Torr, compared to the SS‐MBE ones, which utilizes ultrahigh vacuum in the range of 10^−9^ to 10^−10^ Torr. This makes RP‐CVD epitaxy more robust and economical and thus provides a credible path to large‐scale fabrication technologies.

### 2.5 Percolation Density

The percolation density, *p*_c_, is another one of the important figures of merit to evaluate quality of high‐mobility materials for quantum research and applications. This parameter characterizes disorder at low carrier density that is very important for the fabrication of uniform quantum devices. The percolation occurs when the Fermi level falls below the average profile of potential fluctuations and, therefore, a continuous current flow becomes very much suppressed.^[^ 32, 33 ^]^

**Figure 4:**
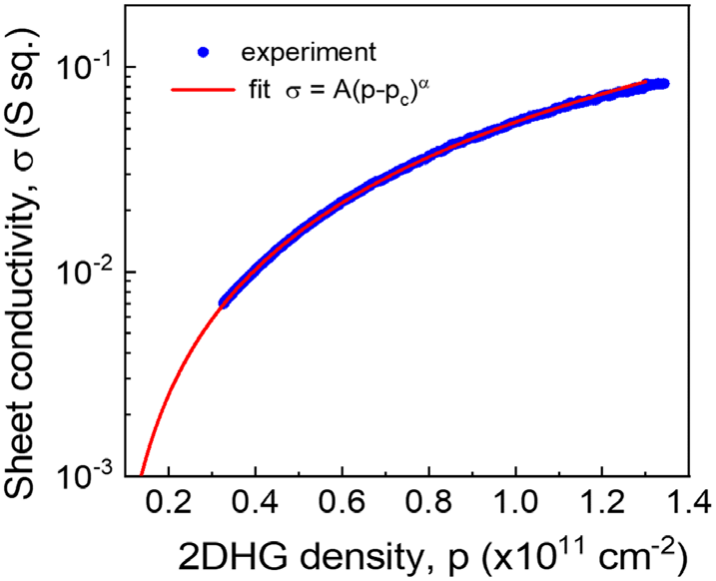
Sheet conductivity versus 2DHG density at 290 mK in linear–log fitted with $\sigma \left(p\right)=A{\left(p-p_{c}\right)}^{\alpha },A=0.059S,p_{c}=5.0\times 10^{9}{\text{cm}}^{-2},\alpha =1.66$.

In **Figure** **4**, we present a plot of conductance at zero magnetic field, $\sigma_{xx}\left(0\right)=1/\rho_{0}$, as a function of carrier density. To estimate *p*_c_, the observed data are then fitted to a percolation conductivity power‐law dependence: $\sigma \left(p\right)\propto {\left(p-p_{c}\right)}^{\alpha }$ with the fitted parameters of α=1.66, and $p_{c}=0.5\times 10^{10}{\text{cm}}^{-2}$ being the critical concentration, that is, the percolation density limit of conductivity. This value is 4 times smaller than the lowest published so far *p*_c_ = 2.1 × 10^10^ cm^−2^ in an undoped s‐Ge QW.^[^ ^30^ ^]^ A detailed study is planned to acquire more physics insights in the percolation properties of this new quantum‐material system at lower temperatures.

### 2.6 Gate Stack Characterization

As shown in Figure 3, we could not reach carrier concentrations larger than 1.8 × 10^11^ cm^−2^ by sweeping the accumulation gate voltage, *V*_G_. At large enough gate voltages, a shift of the percolation threshold voltage occurs, most likely due to a persistent charge accumulation at the dielectric/semiconductor interface. A very similar effect was reported earlier in gated undoped Si/SiGe heterostructures, which was explained as due to a surface tunneling from quantum well to the interface.^[^ ^34^ ^]^ In principle, this effect can be used for the uniformity control of quantum dots in large‐array circuits made from electron Si/Si_1−*x*_Ge_*x*_ or hole Ge/Si_1−*x*_Ge_*x*_ heterostructures.^[^ ^35^ ^]^ More careful investigation of this phenomenon and its applications is outside of the scope of this publication. It should be mentioned that by employing this effect, we have experimentally determined an existence of a stable ***negative*** surface charge of ≈3 × 10^11^ cm^−2^ in the gated Hall‐bar device after initial cool down. This charge is accumulated either at the dielectric–semiconductor interface or within the Al_2_O_3_ gate dielectric. Most likely it is interface charge because we have not observed hysteresis during sweeping gate voltage in both directions in a range less than 0.25 V. This is a relatively large surface charge, comparing to Si/SiO_2_ interface, that could be responsible for the mobility limitation due to scattering on remote ionized impurities. Further planned experiments will provide more insights into the origin of this interface charge and will allow us to either suppress and/or control it. For example, varying thickness of the Si_0.15_Ge_0.85_ cap layer will allow us to understand impact of the dielectric/semiconductor interface on the mobility.

### 2.7 Effective Mass

**Figure 5:**
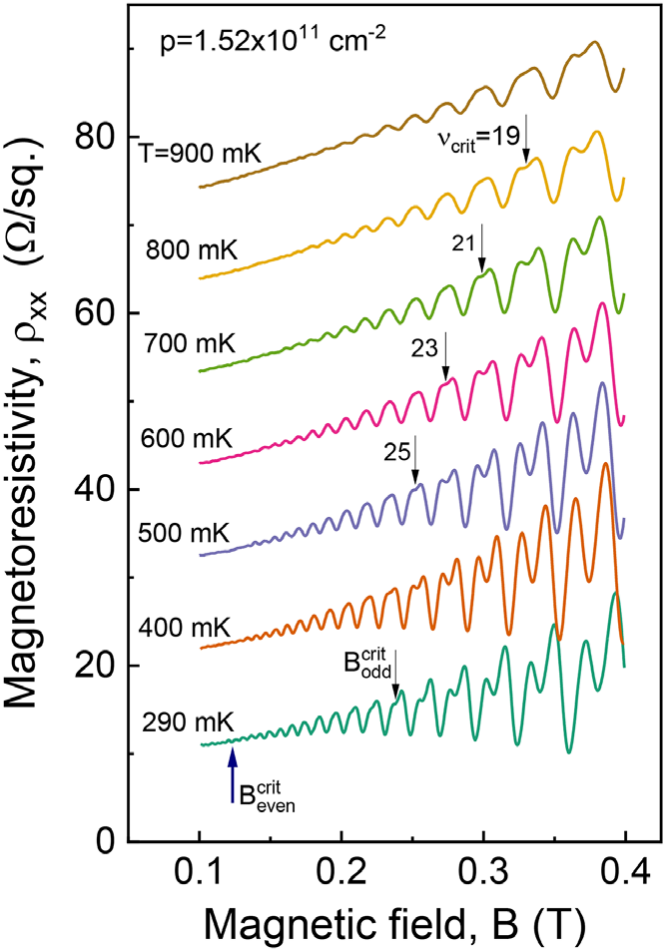
Shubnikov–de Haas traces at different temperatures for *p* = 1.52 × 10^11^ cm^−2^. The critical filling factor when the first spin–split emerge is marked for several traces. Critical magnetic field for odd (spin) and even (cyclotron gap) oscillations is indicated on the lowest trace at base temperature. The amplitude of low‐field oscillations is used to determine hole effective mass, m* = 0.054 *m*_0_.

The effective mass, m*, is another important parameter of a low‐dimensional quantum system. A standard approach to determine the effective mass is to analyze temperature dependence of the Shubnikov–de Haas (SdH) oscillations of magnetoresistivity, $\rho_{xx}$.^[^ ^36^ ^]^ The SdH amplitude is conventionally described by^[^ 36, 37 ^]^ $\Delta \rho_{xx}=4\rho_{0}D_{\text{th}}\left(T\right)\text{exp}\left(-\frac{\pi }{\omega_{c}\tau_{q}}\right)$, where *τ*_*q*_ is the quantum‐scattering time. This expression assumes a Lorentzian density of states (DOS) and corresponds to a “Dingle plot” with ln(Δ*ρ*_*xx*_ */ρ*_0_) to be linear in the *1/B* scale with an intercept (*1/B* = 0) of 4. More generally, the DOS is better described by a Gaussian. If the Gaussian broadening is independent of *B*, the “Dingle plot” becomes proportional to 1/*B* ^2^ with a slope given by (*πΓ/ħω*_c_)^2^. The thermal‐damping term is *D*_th_ = *X*_th_/sinh(*X*_th_), where *X*_th_ = 2*π* ^2^ *k*_B_ *T/E*_gap_. For the simple Landau level (LL) model, without spin‐splitting *E*_gap_ *= ħω*_c_. **Figure** **5** shows the temperature‐dependent traces of SdH oscillations from 290 up to 900 mK for the 2DHG density in the higher density range of 1.52 × 10^11^ cm^−2^. This is a sufficiently large density, and the magnetic fields being sufficiently small, that the oscillations are dominated by the basic cyclotron gap and the standard approach described earlier can be used to estimate hole in‐plane effective mass independent of the LL DOS. Collapsing all the SdH amplitude data in Figure 5 using only one adjustable parameter m* is found that the best fit occurs for an effective hole mass m* = 0.054 *m*_0_. This value is similar to the one reported before in an s‐Ge QW of similar strain along <110> in‐plane crystallographic orientation.^[^ ^7^ ^]^

### 2.8 Spin Properties and g*‐Factor

**Figure 6:**
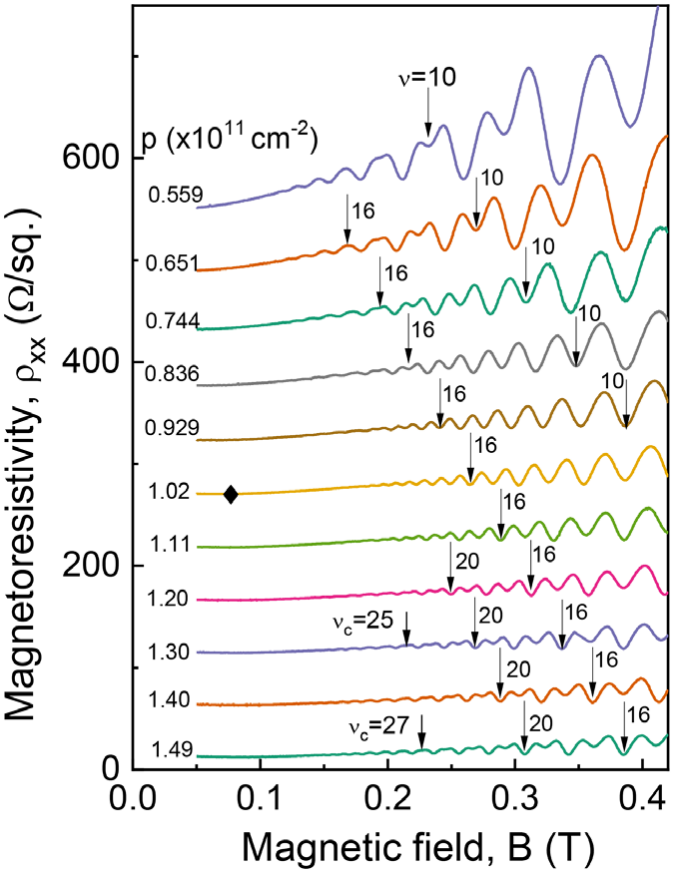
Shubnikov–de Haas traces for different hole densities, *T* = 290 mK. Even minima with corresponding filling factors, *v*, for selected traces are marked by arrows. One trace is marked by a diamond sign which corresponds to the situation when even and odd minima are equal. Critical filling factors, *v*_c_, are marked for two traces, *p* = 1.49 and 1.30 $\times 10^{11}$ cm^−2^.

Information about the spin properties of the 2DHG can be deduced from the SdH oscillations in the normal to the surface magnetic fields. **Figure** **6** shows SdH traces for different hole densities at the base temperature, *T* = 290 mK. Next, we estimate the quantum‐scattering time, *τ*_q_, and quantum mobility, $\mu_{q}=e\tau_{q}/m^{*}$. For this purpose, we use the highest 2DHG density trace in Figure 6 (lower trace, *p* = 1.49 × 10^11^ cm^−2^) because the higher density trace exhibits standard behavior, with the even minima prevailing over the odd (spin) minima in low fields, that is convenient for the analysis. In small magnetic fields, disorder suppresses the SdH oscillations and the amplitude varies as exp(−*π/ω*_c_ *τ*_q_). Using the standard Dingle‐plot analysis^[^ 37, 38 ^]^ for $p=1.49\times 10^{11}{\text{cm}}^{-2}$, we obtain *μ*_q_ = 4.3 × 10^4^ cm^2^ V^−1^ s^−1^ and *τ*_q_ = 1.3 ps. This value is two orders of magnitude smaller than the transport mobility (see **Table** **1**) indicating that the small‐angle scattering dominates in the scattering microscopic processes. The remaining small‐angle scattering mechanisms are generally caused by long‐range Coulomb interactions of mobile carriers with remote ionized impurities or charge dipoles in doping or buffer layers.^[^ 37, 38 ^]^ It is in excellent agreement with our earlier conclusions made independently from the analysis of the 2DHG mobility versus 2DHG density plotted in Figure 3. But in our case, remote impurities are located in the gate stack of the Hall‐bar.

**Table 1:** Summary of 2DHG and 2DEG mobilities and other parameters in s‐Ge, s‐Si, and GaAs low‐dimensional systems

Material	Crystallographic orientation	Mobility, *μ* [×10^6^ cm^2^ V^−1^ s^−1^]	Carrier density, *p* [10^11^ cm^−2^]	Critical density, *p*_c_ [10^10^ cm^−2^]	Effective mass, m* [m_0_]	Dingle ratio, *τ*_t_ */τ*_q_	Transport scattering time, *τ*_*t*_ [ps]	Quantum scattering time, *τ*_q_ [ps]	Effective g*‐factor
2DHG in undoped s–Ge ^[this work]^	<110>	4.3	1.8	0.5	0.054	**100**	**132**	1.3	16‐21
2DHG in MOD s–Ge^[^ ^7^ ^]^	<110>	1.288	2.7	N/A	0.055	60	40.3	0.67	
2DHG in MOD s–Ge^[^ ^7^ ^]^	<100>	1.16	2.7	N/A	0.035	70	23.1	0.33	
2DHG in undoped s–Ge^[^ ^30^ ^]^	No data	≈1	≈1	5	0.068	No data	No data	No data	13.95
2DEG in undoped s–Si^[^ ^28^ ^]^ 50 mK	No data	2.4	≈1	No data	–	No data	No data	No data	No data
2DHG in MOD GaAs^[^ ^55^ ^]^	VdP device	5.8	1.3	N/A	0.3	No data	No data	No data	No data
2DEG in MOD GaAs 300 mK^[^ ^56^ ^]^	VdP device	44	2.0	N/A	0.067	No data	No data	No data	No data

**Figure 7:**
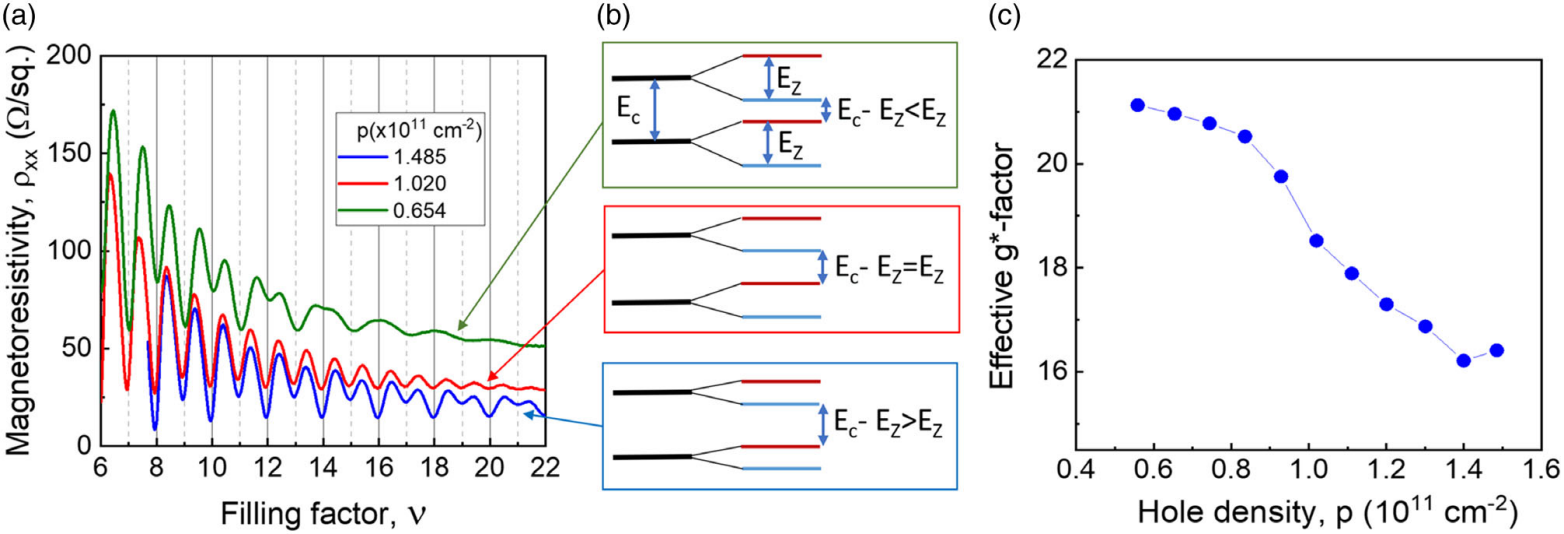
a) Selected Shubnikov–de Haas traces plotted versus filling factor, ν=pℏ/eB. b) Energy diagrams for the corresponding situations: *E*_Z_ > *E*_c_/2 (upper), *E*_Z_ = *E*_c_/2 (middle), and *E*_Z_ < *E*_c_/2 (lower trace). c) Estimated effective hole g‐factor using critical magnetic field for even and odd filling factor oscillations $B_{\text{odd}}^{\text{crit}}$ and $B_{\text{even}}^{\text{crit}}$, assuming Gaussian width of Landau levels, $\Gamma \approx \sqrt{B}$.

Let us continue examination of the SdH oscillations shown in Figure 6 in more details starting from the bottom, the high‐density end. As the density is reduced, the critical filling factor *ν*_c_ decreases and the corresponding critical magnetic field, *B*_crit_, moves to lower fields (indicted in Figure 6 for traces 1.49 and 1.30). It should be emphasized, as can be seen from the labeling in Figure 6, the dominance of the even minima disappears and at the lowest densities, the odd minima are clearly the strongest. At *p* = $1.02\times 10^{11}{\text{cm}}^{-2}$ (marked by a solid diamond in Figure 6), both odd and even minima have equal amplitudes. This is a very special situation indicating that the Zeeman spin splitting (*E*_Z_ = *g* * *μ*_B_ *B*) is equal to the effective cyclotron splitting (reduced by *E*_Z_, i.e., $E_{c}^{*}=\hbar \omega_{c}-E_{Z}$, that means *E*_Z_ = *ħω*_c_/2). This is schematically illustrated in **Figure** **7**b. At this special density, the effective g‐factor is given by g * m*/*m*_*0*_ = 1. Using m*= 0.054 m_0_, we find g* = 18.5. A similar “coincidence” method with tuneable ratio *E*_Z_/*E*_c_, but using tilted magnetic field has been used in electronic structures. For example, in Ref. [39], several coincidence points were observed, *E*_Z_/*E*_c_ = *r*, with *r* = 1/2,1,3/2,2,3 and used to determine the effective electron g*‐factor in a InAs QW structure. For 2DHG structures with strong 2D character, the tilted‐field method does not work in principle due to the strong g‐factor anisotropy of the 2D holes with the in‐plane g*‐factor being close to zero.^[^ 6, 40, 41, 42 ^]^

It should be emphasized that in 2D electron and hole systems, there is a strong electron–electron (hole–hole) exchange interaction that leads to a large g*‐factor enhancement.^[^ ^43^ ^]^ In particular for materials with small effective g*‐factor like GaAs, this enhancement effect dominates the Zeeman splitting.^[^ ^44^ ^]^ However, the exchange enhancement requires a large spin polarization of the LLs, which is suppressed at low fields by the disorder damping, so the observation of the large splitting, *E*_Z_ = *ħω*_c_/2, persisting to the lowest fields at which oscillations are visible suggests that the splitting is substantially intrinsic in our experiment for the trace marked by a diamond in Figure 6, and not exchange induced. In the literature, the problem of the magnitude of the exchange interaction so far is not well understood.^[^ ^45^ ^]^ It depends simultaneously on several effects involving the LL width, *B*‐dependent filling factor, DOS function, and temperature that requires a careful self‐consistent approach.

To examine more carefully the spin‐cyclotron gap coincidence transition discussed earlier, Figure 7a shows SdH oscillations for 3 selected 2DHG densities plotted versus filling factor, *ν = pħ/*e*B*. The SdH trace at high hole density of 1.485 × 10^11^ cm^−2^ in Figure 7a presents a normal situation of SdH oscillations when even minima prevail. However, at the lowest density of 0.654 × 10^11^ cm^−2^ (upper trace), the situation is clearly reversed: the odd minima corresponding to the spin gaps are much stronger than the even ones. The middle trace (*p* = 1.02 × 10^11^ cm^−2^) corresponds to the special “coincidence” situation when odd and even minima have the same amplitude at the whole magnetic‐field range. In Figure 7b, we show the qualitative energy diagrams corresponding to the three situations when the spin gap is smaller than half of the cyclotron gap, *E*_Z_ < *E*_c_/2, when *E*_Z_ = *E*_c_/2 (the middle trace), and when *E*_Z_ > *E*_c_/2 (the upper trace). The SdH oscillations are periodic in the inverse magnetic field with minima at digital filling factors, *ν = pħ/*e*B*, as is evident in Figure 7a. At low fields, when the spin splitting is not resolved, the period of the oscillation versus the filling factor, *ν,* increases by 2 for each oscillation. It can be seen in the upper trace in Figure 7 in the range ν>14. At some critical field (marked as $B_{\text{odd}}^{\text{crit}}$ in Figure 5 and as *v*_*c*_ in Figure 6), the spin splitting starts to be resolved and the period of SdH oscillations Δ*ν* is reduced to one (see Figures 5 and 6). Spin minima become more pronounced with increasing magnetic field and appear between the peaks corresponding to even filling factors. For the normal situation in Figure 6 and for $p>1.02\times 10^{11}{\text{cm}}^{-2}$, the cyclotron minima, at even filling factors, remain deeper than the spin minima indicating the cyclotron gap is larger than the spin gap.

Experimentally, a linear “Dingle” plot $\ln\left(\frac{\Delta \rho_{xx}}{\rho_{0}}\right)$ vs 1/*B* (not shown) indicates Lorentzian broadening of the LLs, Γ ≈ (exp(−*π*/*ω*_c_ *τ*_q_), or equivalently Gaussian with a broadening Γ that increases as *B* ^1/2^. Frequently, however, the Dingle plot shows a quadratic dependence on 1/*B* indicating a broadening with constant Gaussian width Γ at least over the measurement range of fields.^[^ ^46^ ^]^ Furthermore, while Γ may increase as *B* ^1/2^ at higher fields, the self‐consistent Born approximation suggests that it also decreases as the spins become resolved.^[^ ^47^ ^]^ This competition between the two factors means that it is not clear how the broadening actually varies with the magnetic field. A simple approximation for estimating effective g*‐factor^[^ ^48^ ^]^ is to use the two critical fields $B_{\text{odd}}^{\text{crit}}$ and $B_{\text{even}}^{\text{crit}}$ when the respective minima first occur and assume *E*z/Γ and (*ħω*_c_–*E*_Z_)/*Γ* take the same value at these two fields. Assuming Γ ≈ *B* ^1/2^, then gives g* = *E*_Z_/*μ*_B_ *B* = 2(m*/m_0_)√*B*_even_/(√*B*_even_ + √*B*_odd_). The results of this approximation are shown in Figure 7c. A more detailed analysis (to be published elsewhere) without any specific *B*‐dependence of Γ, but assuming that it is the same for maxima and minima at each field gives similar values.

Estimations of the effective g*‐factor at different densities can be obtained from the analysis of the LL DOS, presented in Figure 7. If we assume LLs are described by Gaussians, then the odd minima are characterized by DOS with an exp(−*E*_z_^2^/4Γ^2^) dependence and the even minima being proportional to exp(−(*E*_Z_–*ħω*_c_)^2^/Γ^2^). The *B*‐dependence of the minima should then reveal *E*_Z_ provided the values of the LL width, Γ, are known. This is not a straightforward problem, although, it might appear that the conventional exp*(−π/ωτ*_q_) amplitude dependence gives a value of Γ that apparently varies as *B* ^1/2^. The analysis confirms the simple prediction, g* ≈ 18, with a small downward trend with increasing density. However, there is no reason to believe that the values of effective spin gaps, $E_{Z}^{*}$ (with accounted exchange interaction), are the same for the even and odd minima. It is well known that for exchange enhancement,^[^ 44, 45 ^]^ there is a strong oscillatory dependence of the effective enhanced spin splitting, $E_{Z}^{*}$. Nevertheless, a good gate‐voltage tunability of the effective g*‐factor with the gate voltage (density) is evident in Figure 7c, within a range that is more than sufficient for the quantum‐computing applications. Note that an excessively large g*‐factor voltage‐sensitivity may result in undesirable increased spin qubit noise figures due to capacitive coupling to control gates. An apparent nonlinear dependence of g*‐factor in Figure 7c may be partially due to the exchange interaction discussed therein.^[^ 43, 44, 49 ^]^ A more detailed analysis of this complex self‐consistent mechanism of the exchange interaction is outside of this paper.

### 2.9 Comparison with the State of the Art

It is well known that electron g*‐factor in GaAs is very small $g_{GaAs}^{*}\approx -0.44$; for holes, it is around 1.4.^[^ ^50^ ^]^ In Si, effective g*‐factor for both electrons and holes is around 2.^[^ 51, 52 ^]^ In III–V materials, InAs and InSb, electron g*‐factor reaches relatively high values of ≈−15^[^ ^39^ ^]^ and up to −50,^[^ ^53^ ^]^ correspondingly. Unfortunately, III–V materials are very complicated to process, very expensive, not widely abundant in the earth crust compared to Si, do not exist in isotopically pure forms, and are not compatible with the state‐of‐the‐art Si technologies for mass production. Therefore, the obtained results make the s‐Ge on Si material very attractive for further research of quantum physics and for development of novel quantum technologies.

Low‐temperature 2DHG properties obtained in this work are summarized in the Table 1. For comparison, the highest 2DHG mobilities obtained in MOD and undoped s‐Ge QW are shown as well. For completeness, the highest mobilities of 2DEG in s‐Si and GaAs and 2DHG in GaAs are added to the table (three last rows). Material's structures along with the corresponding references are listed in the first column. The crystallographic orientation of the Hall‐bar (when applicable) is specified in the second column. It was found that transport mobility strongly depends on the Hall‐bar orientation in s‐Ge QW structures.^[^ ^7^ ^]^ The maximum low‐temperature carrier mobility is quoted in the third column along with the corresponding concentration shown in the fourth column. The highest mobility so far is obtained for 2DEG in GaAs, 44 × 10^6^ cm^2^ V^−1^ s^−1^. We believe that hole mobility in s‐Ge can reach similar values or even higher due to the record‐small hole effective mass in this material system, which can be engineered using compressive biaxial strain in the Ge epilayer of very low disorder and smooth interfaces. Dingle ratio of the scattering times $\tau_{t}/\tau_{q}$ is given in the seventh column. As expected for high‐mobility samples, it is very large and reaches 100 in s‐Ge in this work. The large Dingle ratio indicates a very strong dominance of small‐angle scattering mechanisms that limit quantum‐scattering time as discussed earlier. It will be important to study and identify exact microscopic scattering mechanisms in s‐Ge QWs for further advances in quality of this material platform. Both transport‐ and quantum‐scattering times are shown in the eighth and ninth columns, respectively. The last column shows available g*‐factor data in s‐Ge. Unfortunately, there are no g*‐factor data in the quoted works cited in this table. Effective g*‐factor values in other materials are discussed in Section 2.8.

As a last note, this work reduces the gap between the best 2DHG mobility in GaAs QW heterostructures grown on GaAs substrate, which was recently increased from 2.3 × 10^6^ cm^2^ V^−1^ s^−1^ (at carrier density 6.5 × 10^10^ cm^−2^)^[^ ^54^ ^]^ to 5.8 × 10^6^ cm^2^ V^−1^ s^−1^ (1.3 × 10^10^ cm^−2^), measured at 300 mK.^[^ ^55^ ^]^ All other known semiconductors, including III–V, II–VI, perovskites, 2D materials, etc., show substantially lower 2DHG mobility than in the s‐Ge and GaAs QW structures. Moreover, it is important to emphasize that s‐Ge QWs are grown on standard Si(001) wafers, which are used by the semiconductor industry to fabricate over 99% of all modern electronic devices including complementary metal oxide semiconductor (CMOS) devices. RP‐CVD in particular enables epitaxial growth of these QW structures on 300 mm diameter wafers now and can be extended to larger 450 mm wafers in near future. This is an additional attractive feature of the reported s‐Ge material system for large‐scale applications.

## 3 Conclusions

A record‐high mobility of free holes reaching 4.3 × 10^6^ cm^2^ V^−1^ s^−1^ in strained germanium grown on a standard silicon wafer has been demonstrated that sets a new quality benchmark for the group IV semiconductor materials. As a consequence, electrons are outperformed by holes in the group IV semiconductor materials at low temperatures. The demonstrated hole mobility in s‐Ge is twice that of the best mobility of electrons reported in state‐of‐the‐art strained silicon. A similar situation has not been observed for any other semiconductor material. Due to the fourfold material quality improvement, it can be stated that the novel class of quantum materials for the quantum‐physics research and applications has emerged. This superior material system with a combination of unique properties, which are large and tuneable effective g*‐factor, strong and tuneable SOI, low percolation density, and small effective mass, will lead to new opportunities for innovative quantum‐device technologies and applications in quantum as well as in classical electronics, optoelectronics, and sensors.

## Conflict of Interest

The authors declare no conflict of interest.

## Author Contributions

M.M. developed the CVD growth technology and prepared the s‐Ge epi wafers, M.M. and S.S. designed the experiment and wrote the article with contributions from J.K. and P.C., S.S. and J.K. conducted the experiment, S.S. and P.C. analyzed transport data and spin effects; A.B. developed automatization of the experiment and software support; P.W., W.H. and P.B. developed the fabrication technology and fabricated the gated Hall bar device.

## Data Availability

The data that support the findings of this study are available from the corresponding author upon reasonable request.
